# EUS-guided gastrojejunostomy for management of malignant gastric outlet obstruction in a patient with Roux-en-Y anatomy

**DOI:** 10.1016/j.vgie.2022.06.001

**Published:** 2022-07-21

**Authors:** Charlotte Campbell, Rishi Pawa

**Affiliations:** Wake Forest School of Medicine, Winston-Salem, North Carolina

**Keywords:** EUS-GJ, endoscopic ultrasound-guided gastrojejunostomy, GJ, gastrojejunostomy, LAMS, lumen-apposing metal stent, NC, nasocystic

## Abstract

Video 1 Endoscopic ultrasound–guided gastrojejunostomy.

Endoscopic ultrasound–guided gastrojejunostomy.

## Background

Gastric outlet obstruction is a complication of advanced gastrointestinal malignancies and contributes significantly to patient morbidity. Surgical gastrojejunostomy (GJ) and enteral stenting have been traditionally employed for management in these patients. Endoscopic ultrasound–guided gastrojejunostomy (EUS-GJ) with a lumen-apposing metal stent (LAMS) provides an alternative to luminal stenting and surgical GJ. We present a case of EUS-GJ performed in a patient with Roux-en-Y anatomy.

## Case Report

**Figure 1:**
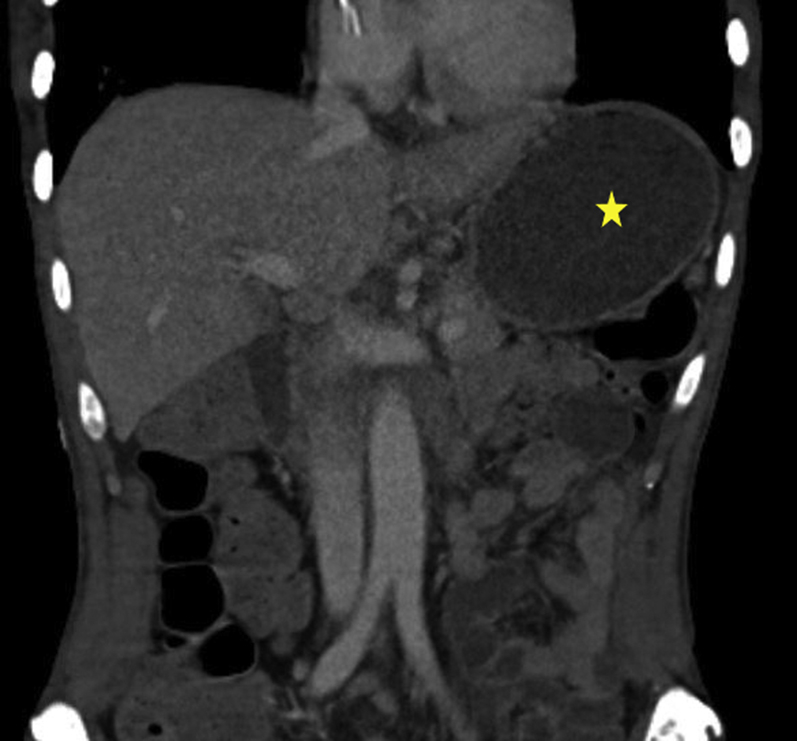
CT scan of abdomen showing a distended gastric pouch (*yellow asterisk*) secondary to gastric outlet obstruction.

**Figure 2:**
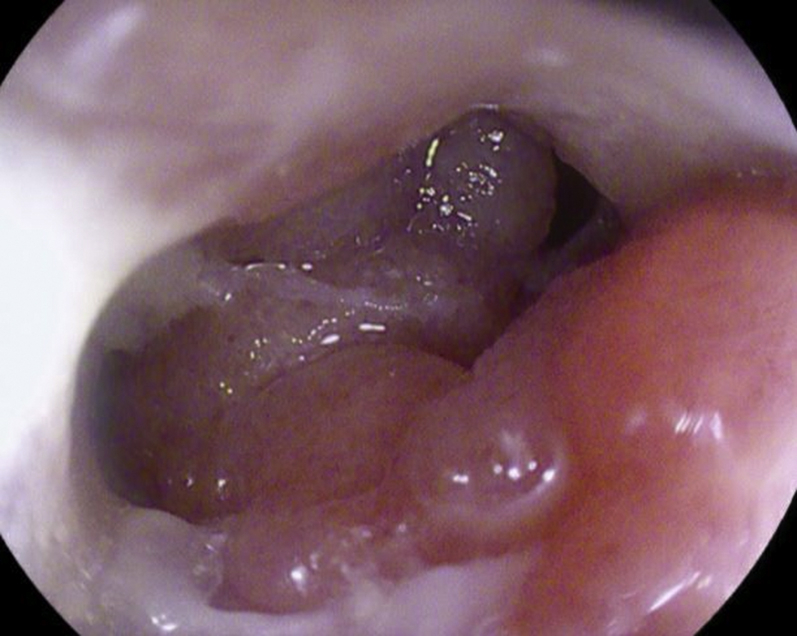
Gastrojejunal anastomotic stenosis secondary to tumor infiltration.

**Figure 3:**
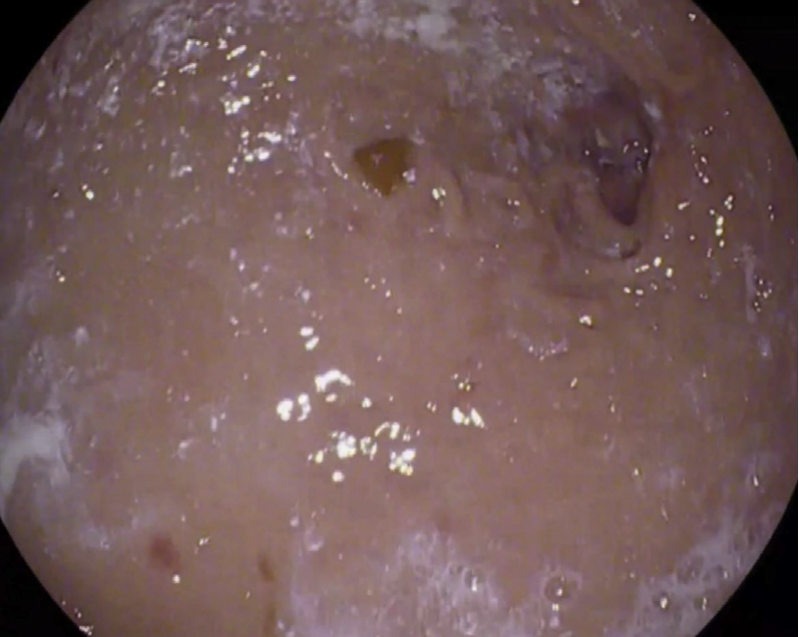
Distended gastric pouch with normal appearing mucosa.

**Figure 4:**
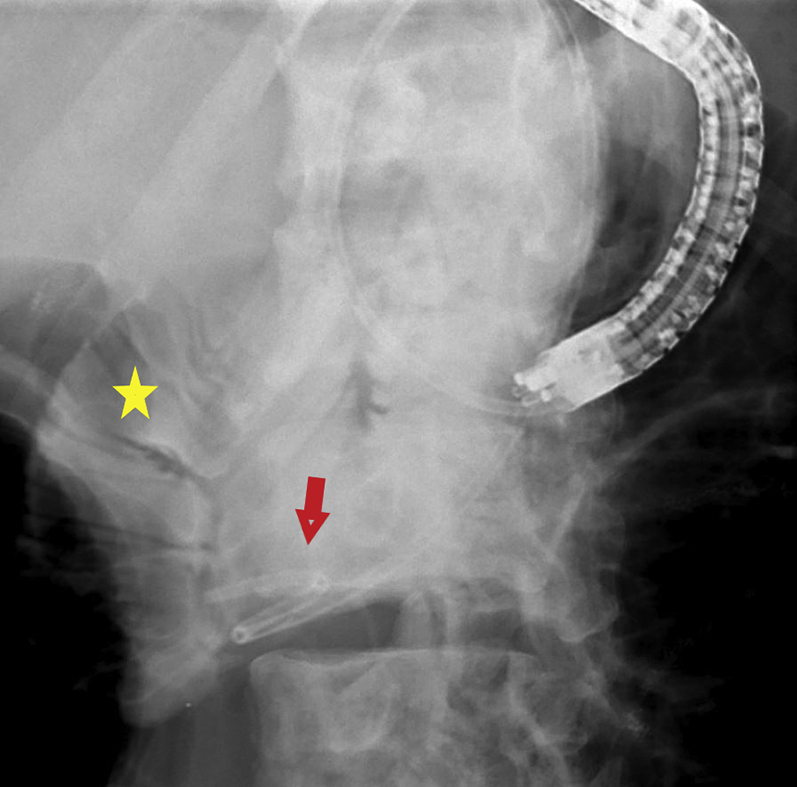
Fluoroscopic image showing contrast in the small bowel (*yellow asterisk*) and nasocystic drain in the proximal jejunum (*red arrow*).

**Figure 5:**
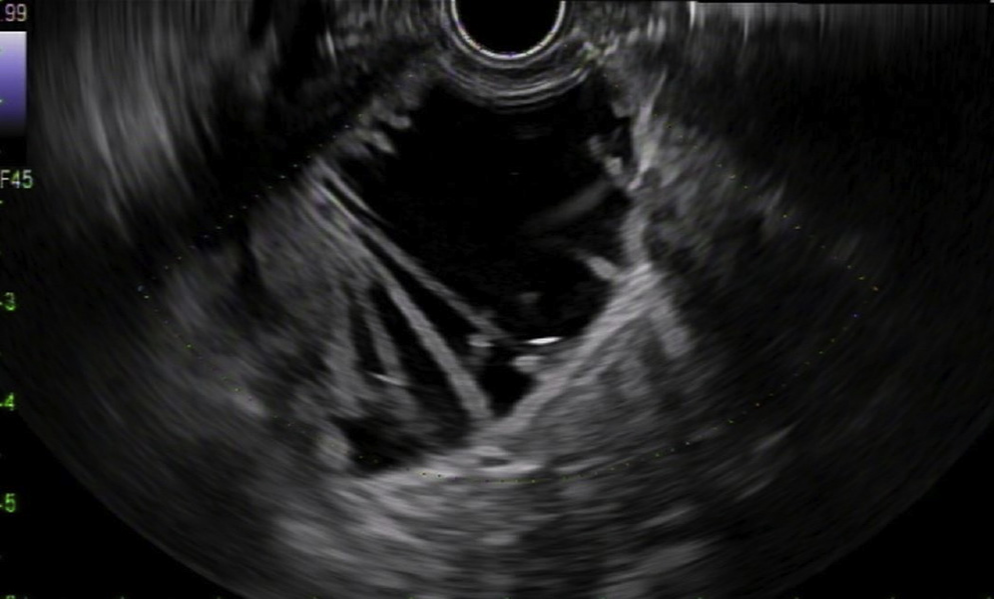
Endosonographic visualization of contrast filled jejunal limb for creation of gastrojejunostomy.

**Figure 6:**
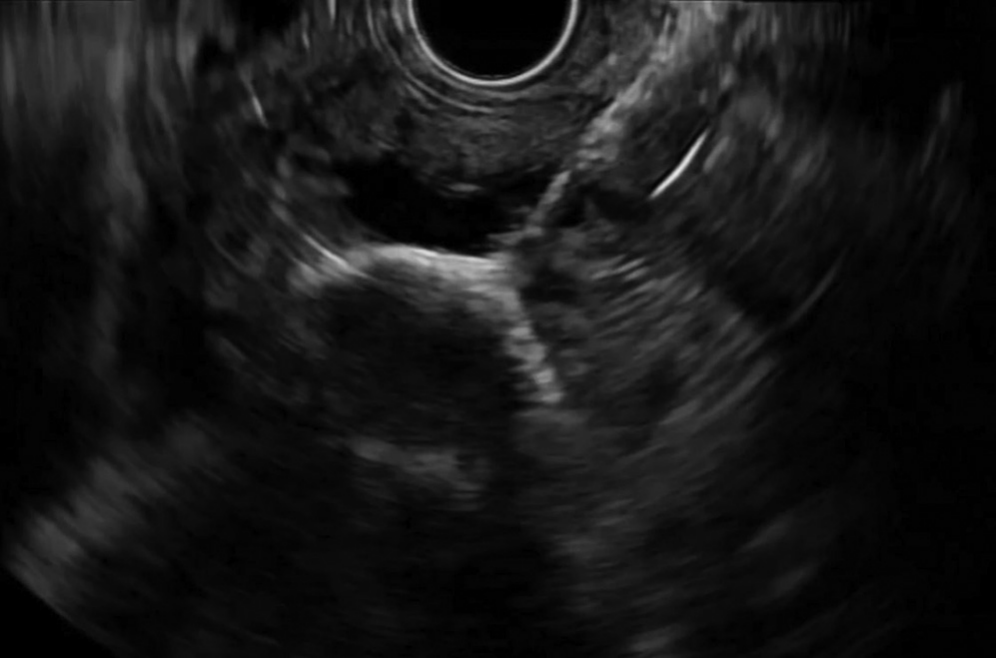
Endosonographic image showing distal flange of lumen-apposing metal stent deployed in the jejunal limb.

**Figure 7:**
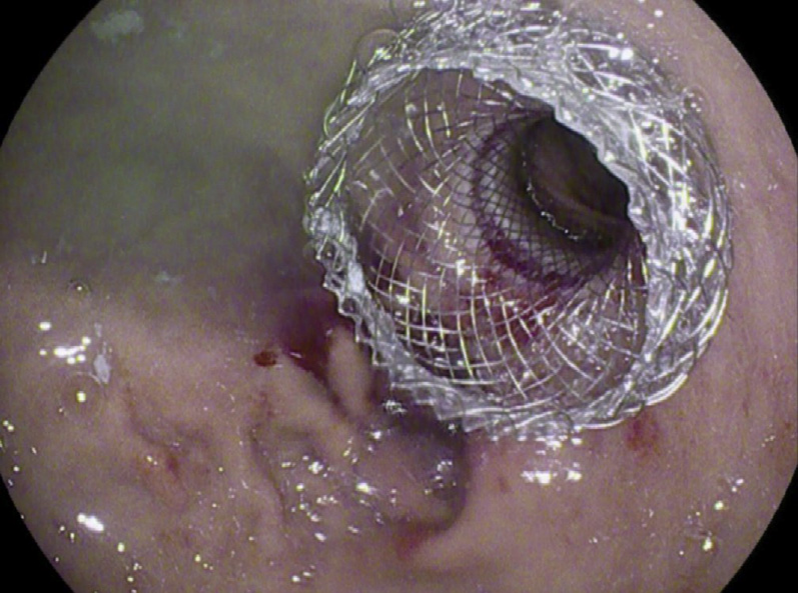
Endoscopic image following deployment of lumen-apposing metal stent for creation of gastrojejunostomy.

**Figure 8:**
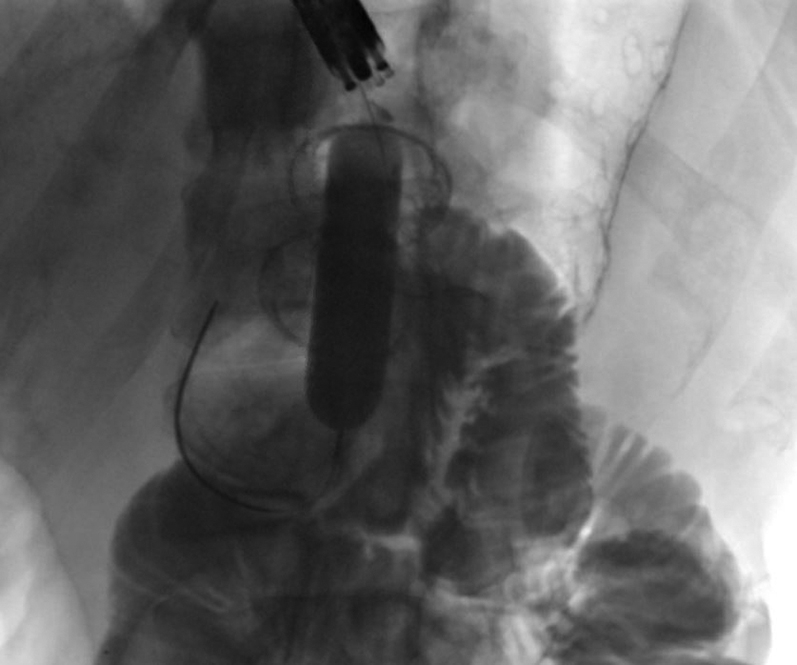
Fluoroscopic image showing balloon dilation of lumen-apposing metal stent.

**Figure 9:**
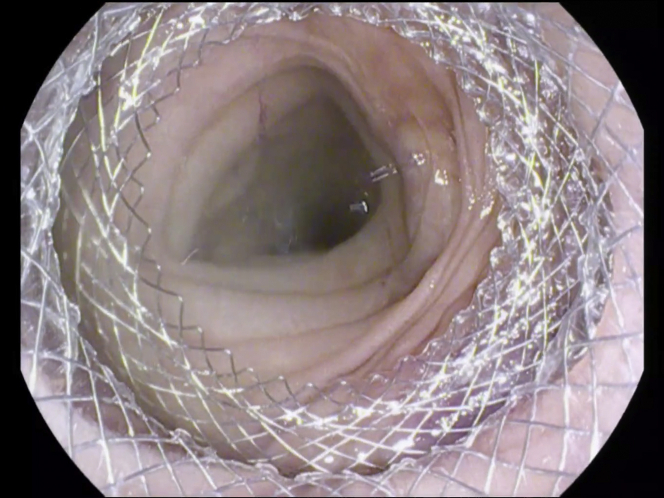
Endoscopic image showing successful creation of gastrojejunostomy with visualization of healthy jejunal mucosa.

A 56-year-old man was transferred from an outside hospital for management of gastric outlet obstruction. His medical history was significant for metastatic duodenal adenocarcinoma, status postpartial gastrectomy, and duodenectomy with Roux-en-Y GJ. Endoscopy done at the outside hospital revealed an ulcerated stricture at the gastrojejunal anastomosis, with biopsies confirming recurrent adenocarcinoma. CT scans of the abdomen and pelvis showed postsurgical changes of Roux-en-Y GJ. The proximal stomach was distended with an air-fluid level (Fig. 1). After discussion with the patient, the decision was made to proceed with EUS-GJ (Video 1, available online at www.giejournal.org). Upon endoscopy, a high-grade stricture was visualized at the gastrojejunal anastomosis (Fig. 2). The gastric pouch appeared distended measuring 6 to 7 cm in length (Fig. 3). Using a sphincterotome preloaded with a 0.025-inch-diameter guidewire, the small bowel was selectively intubated. Contrast was injected to opacify the proximal jejunum. Following advancement of the guidewire deep into the jejunum, a 7-Fr nasocystic (NC) catheter was passed over the guidewire (Fig. 4). The gastroscope was removed leaving the NC catheter in place. The NC catheter was attached to a water jet allowing infusion of diluted contrast mixed with methylene blue into the small bowel. With the echoendoscope positioned in the gastric pouch, a dilated small bowel loop was visualized (Fig. 5). Under EUS guidance, the small bowel was punctured with a 22-gauge needle through an area of the gastric pouch free from cancer. Methylene blue was aspirated, confirming appropriate needle position in the jejunum. Maintaining the same EUS view, direct placement of a 20-mm electrocautery enhanced LAMS was performed. The distal flange was deployed under EUS guidance (Fig. 6). Following deployment of the proximal flange, bluish colored fluid was seen draining from the stent, confirming appropriate stent position in the jejunum. The echoendoscope was then exchanged with a forward-viewing gastroscope with visualization of LAMS in the gastric pouch, proximal to the stenotic anastomosis (Fig. 7). Using a wire-guided balloon, the LAMS was dilated to 15 mm under endoscopic and fluoroscopic guidance (Fig. 8). Following dilation, the endoscope was advanced through the LAMS into the small bowel revealing healthy jejunal mucosa (Fig. 9). A CT scan obtained the following day showed appropriate LAMS position and decreased gastric distension with oral contrast opacifying the stomach and small bowel. The patient was started on a clear liquid diet and gradually advanced to a soft mechanical diet over 24 hours. At the 4-week follow-up, he was tolerating a regular diet without nausea and vomiting and had gained 12 pounds since the procedure. The current plan is to leave the existing stent in place for 6 months, at which time the patient will be scheduled for LAMS exchange.

## Conclusions

The off-label use of LAMS for management of malignant gastric outlet obstruction has continued to evolve. In comparison with enteral stenting, EUS-GJ has lower rates of stent failure and symptom recurrence requiring reintervention.^1^^,^^2^ EUS-GJ has a similar clinical efficacy to laparoscopic GJ with fewer adverse events, lower costs, and shorter hospital stays.^3^^,^^4^ Technical failure and adverse events associated with EUS-GJ are commonly related to stent misdeployment.^5^ Increased familiarity with this technique has been shown to mitigate these complications.

## Disclosure

*The authors disclosed no financial relationships.*

## Supplementary data

Video 1 Endoscopic ultrasound–guided gastrojejunostomy.
